# Frequent Mobile Electronic Medical Records Users Respond More Quickly to Emergency Department Consultation Requests: Retrospective Quantitative Study

**DOI:** 10.2196/14487

**Published:** 2020-02-14

**Authors:** Kwang Yul Jung, SuJin Kim, Kihyung Kim, Eun Ju Lee, Kyunga Kim, Jeanhyoung Lee, Jong Soo Choi, Mira Kang, Dong Kyung Chang, Won Chul Cha

**Affiliations:** 1 Department of Emergency Medicine Inha University School of Medicine Incheon Republic of Korea; 2 Department of Digital Health Samsung Advanced Institute for Health Science & Technology Sungkyunkwan University Seoul Republic of Korea; 3 Korea Health Industry Development Institute Cheongju Republic of Korea; 4 Statistics and Data Center Research Institute for Future Medicine Samsung Medical Center Seoul Republic of Korea; 5 Health Information and Strategy Center Samsung Medical Center Seoul Republic of Korea; 6 Center for Health Promotion Samsung Medical Center Sungkyunkwan University School of Medicine Seoul Republic of Korea; 7 Department Gastroenterology Samsung Medical Center Sungkyunkwan University School of Medicine Seoul Republic of Korea; 8 Department of Emergency Medicine Samsung Medical Center Seoul Republic of Korea

**Keywords:** electronic medical record, emergency department, mobile health, time efficiency

## Abstract

**Background:**

Specialty consultation is a critical aspect of emergency department (ED) practice, and a delay in providing consultation might have a significant clinical effect and worsen ED overcrowding. Although mobile electronic medical records (EMR) are being increasingly used and are known to improve the workflow of health care providers, limited studies have evaluated their effectiveness in real-life clinical scenarios.

**Objective:**

For this study, we aimed to determine the association between response duration to an ED specialty consultation request and the frequency of mobile EMR use.

**Methods:**

This retrospective study was conducted in an academic ED in Seoul, South Korea. We analyzed EMR and mobile EMR data from May 2018 to December 2018. Timestamps of ED consultation requests were retrieved from a PC-based EMR, and the response interval was calculated. Doctors’ log frequencies were obtained from the mobile EMR, and we merged data using doctors’ deidentification numbers. Pearson’s product-moment correlation was performed to identify this association. The primary outcome was the relationship between the frequency of mobile EMR usage and the time interval from ED request to consultation completion by specialty doctors. The secondary outcome was the relationship between the frequency of specialty doctors’ mobile EMR usage and the response time to consultation requests.

**Results:**

A total of 25,454 consultations requests were made for 15,555 patients, and 252 specialty doctors provided ED specialty consultations. Of the 742 doctors who used the mobile EMR, 208 doctors used it for the specialty consultation process. After excluding the cases lacking essential information, 21,885 consultations with 208 doctors were included for analysis. According to the mobile EMR usage pattern, the average usage frequency of all users was 13.3 logs/day, and the average duration of the completion of the specialty consultation was 51.7 minutes. There was a significant inverse relationship between the frequency of mobile EMR usage and time interval from ED request to consultation completion by specialty doctors (coefficient=–0.19; 95% CI –0.32 to –0.06; *P*=.005). Secondary analysis with the response time was done. There was also a significant inverse relationship between the frequency of specialty doctors’ mobile EMR usage and the response time to consultation requests (coefficient=–0.18; 95% CI –0.30 to –0.04; *P*=.009).

**Conclusions:**

Our findings suggest that frequent mobile EMR usage is associated with quicker response time to ED consultation requests.

## Introduction

### Background

Specialty consultation is a critical aspect of emergency department (ED) practice [1]. Specialty consultation occurs when an ED doctor contacts a specialty doctor to decide the disposition of patients who require care beyond the scope of ED services. There are five types of specialty consultations: admission, seeking opinion, special procedures, transfer of care, and outpatient referrals [2].

A delayed response from the specialty doctor can cause disposition delay and contribute to ED crowding, which has been a critical public health concern [1,3-6]. It can also result in conflicts and misunderstanding among doctors, which can even lead to life-threatening outcomes for the patients and legal problems for the doctors [1]. Although many studies have suggested ways to manage consultation delays [2,7,8], consultation is still a laborious task. Strategies to reduce consultation delay should be considered and implemented.

### Mobile Technology

Mobile electronic medical records (EMR) are being increasingly used by health care providers [9-11]. Several studies have reported that ubiquitous access to patient data through mobile EMRs and their portability in real-time can help doctors work more efficiently [12,13]. Studies conducted in EDs with mobile devices or mobile EMRs showed positive results for the improvement of clinical flow and efficiency [14-16]. However, given that mobile EMR usage has been generally low and varies among doctors [10,17,18], most studies evaluated the impact of mobile EMR using surveys or simulation methods [12,14-16]. To the best of our knowledge, none of the previous studies evaluated time efficiency in a real clinical setting for the frequency of mobile EMR usage.

### Study Objectives

In this study, we aimed to determine the association between doctor’s response duration to ED consultation requests and their frequency of mobile EMR use.

## Methods

### Study Setting

This retrospective study was conducted in the ED of Samsung Medical Center, Seoul, South Korea. The ED is part of a tertiary academic teaching hospital with 2000 inpatient beds and daily average outpatient visits of more than 9000 in 2018. The ED consists of 69 treatment beds and admits an average of 200 patients daily. The total number of ED cases in 2018 was approximately 79,000. The study protocol was reviewed and approved by the Samsung Medical Center Institutional Review Board (IRB #2019-01-113-001).

### Mobile Electronic Medical Records

The hospital’s EMR system, known as DARWIN (Data Analysis & Research Window for Integrated kNowledge), was launched in July 2016 with both PC and mobile versions. The mobile DARWIN (mDARWIN) is based on Android 2.3 Gingerbread (Google Inc, Mountain View, California, United States) and has Wi-Fi and 3G capabilities. It consists of the main menu, list-level features, and patient-level features. After logging in to the system using their identification number and password, users can select from the main menu to view a list-level feature or select a function. From each list-level feature, users can select patient-level features for more activities or leave and move to other list-level features. Each session closes either when a user logs out or after no activity has taken place for a certain amount of time. The mDARWIN also supports fingerprint log in and near-field communication.

### Specialty Consultation Process

**Figure 1:**
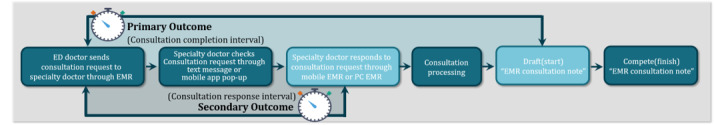
The specialty consultation process flow and outcome measurement of the study. The primary outcome was a measurement of the interval between consultation request and draft consultation note, while the secondary outcome used the interval between consultation request and response. ED: emergency department; EMR: electronic medical record.

**Figure 2:**
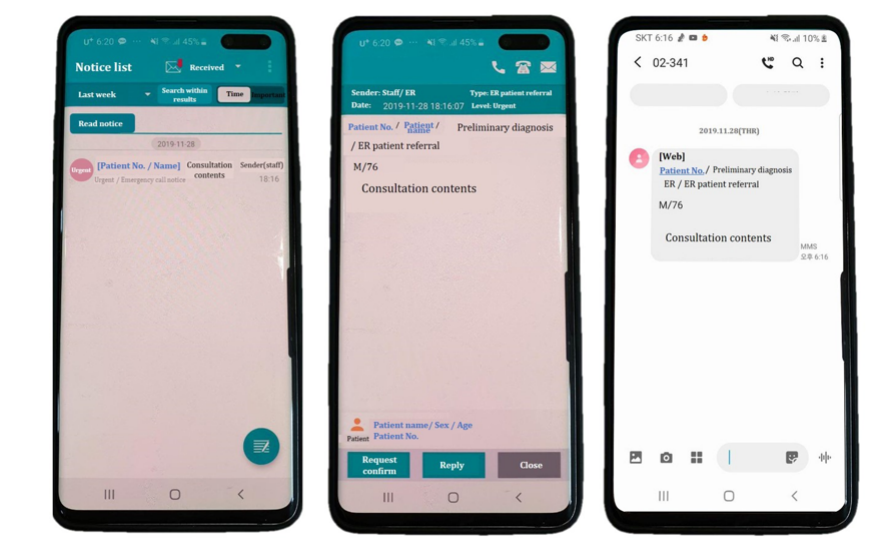
Screenshots of a consultation request made via mobile electronic medical record popup (left, middle) or text message (right).

A specialty consultation involves three necessary steps: (1) sending a request; (2) responding to the request; and (3) answering the request (Figure 1). All consultations are requested through EMR except for the radiology department’s interpretations of imaging findings, which is done via a PC messenger. When an ED doctor makes a specialty consultation request to a specific specialty department in the “Specialty consultation administration” window in the EMR, the written request is immediately sent via a mobile EMR notification and mobile text message to the on-call doctor in the department at the time of consultation request (Figure 2).

The specialty doctor acknowledges the consultation, which is verified by opening the mobile EMR notification or using the nearest PC EMR at that time. Either of these two actions is recorded as a response in the “Specialty consultation administration,” and the ED doctor is notified of the delivery of their consultation to the specialty doctor. The specialty doctor then goes to the ED and sees the patients. After examining the patients, the consultation note from the specialty doctor can be written only via PC-based EMR and not mobile EMR. The timestamp of drafting and completing the consultation note is recorded, and a completed consultation note by the specialty doctor is considered the closing of the consultation. The ED doctor can then make decisions on patient disposition according to the note.

### Study Subjects

We analyzed specialty consultations for patients presented to the ED from May 2018 to December 2018. Patients who left without being examined or patients who were transferred directly to another location, like the delivery room, were excluded. Specialty consultations of radiology, pediatric, and emergency departments, which have different consultation processes, were also excluded. Doctors were categorized based on occupation (staff, fellows, and residents) and specialty (physician, surgeon, or another hospital-based group). The physician group consisted of internal medicine, the surgeon group was comprised of general surgery, neurosurgery, thoracic surgery, otolaryngology, ophthalmology, obstetrics/gynecology, orthopedic surgery, plastic surgery, and the urology department. The other hospital-based groups included critical-care medicine, dentistry, neurology, psychiatry, radio-oncology, and rehabilitation medicine. Consultations lacking time information due to missing consultation notes, or consulting without responses, were excluded.

### Data Collection and Processing

Timestamp data on emergency consultations were retrieved from the EMR of the patients admitted to the ED during the study period. Data on three types of timestamps were collected: (1) time of consultation request by the ED doctor, which is recorded in the PC EMR by the requesting ED physician; (2) time of response made by the specialty doctor, which is recorded in the EMR or mobile EMR by the requested specialty physician; and (3) time of drafting the consultation note by the specialty doctor, which is recorded when the specialty doctor selects “draft consultation note.” Then, the time intervals were calculated, with time interval (1) to (3) defined as the consultation completion interval, and time interval (1) to (2) defined as the consultation response interval.

The frequency of mDARWIN usage was extracted from the log data. Frequency was defined as the sum of all event logs such as log in, selecting an option, log out, and other actions on the mDARWIN. The overall usage of individual features in the mDARWIN was examined by summarizing the usage frequencies of features from the log data. Timestamp data were merged with doctors’ log frequency data using doctors’ deidentification numbers. The merged data were then analyzed.

### Outcome Measures and Sensitivity Analysis

The primary outcome was the relationship between the frequency of mDARWIN usage and the consultation completion interval. The drafting of the consultation note is considered to be the completion of the request. The ED physician then discharges the patients according to the consultation note. If the consultation note by the specialty physician advises further examinations, such as laboratory tests or additional radiological tests, the disposition decision will be delayed until the advised tests have been completed. The interval time from drafting the consultation note to finalizing the consultation note is determined by patient factors rather than workflow. Without a conclusion, answer of disposition, or additional examination of the consulted patient, the specialty physician cannot write a consultation note; the drafting of the consultation note, therefore, indicates the end of the consultation process.

Subanalysis was performed with the group of specialty doctors who had left log records between the timestamp of the ED consultation request and the timestamp for drafting the consultation note. We calculated the median mobile log frequency for this group and compared the median interval time between the high-frequency group (higher than the median frequency) and the low-frequency group (lower than the median frequency).

The secondary outcome was the relationship between the frequency of mDARWIN usage and the consultation response interval. The primary and secondary outcomes are depicted in Figure 1.

### Statistical Analysis

Continuous variables are expressed in terms of averages and standard deviations, and categorical variables are expressed in frequencies and percentages. To identify a correlation between frequency and time intervals, we performed Pearson’s product-moment correlation and analyzed the summary of the linear model. The differences between the groups were examined using a one-tailed Student’s *t* test for categorical variables. *P* values <.05 were considered significant. Data analyses were performed using R software version 3.4.2 (The R Project for Statistical Computing, Vienna, Austria).

## Results

### Characteristics of the Subjects

**Figure 3:**
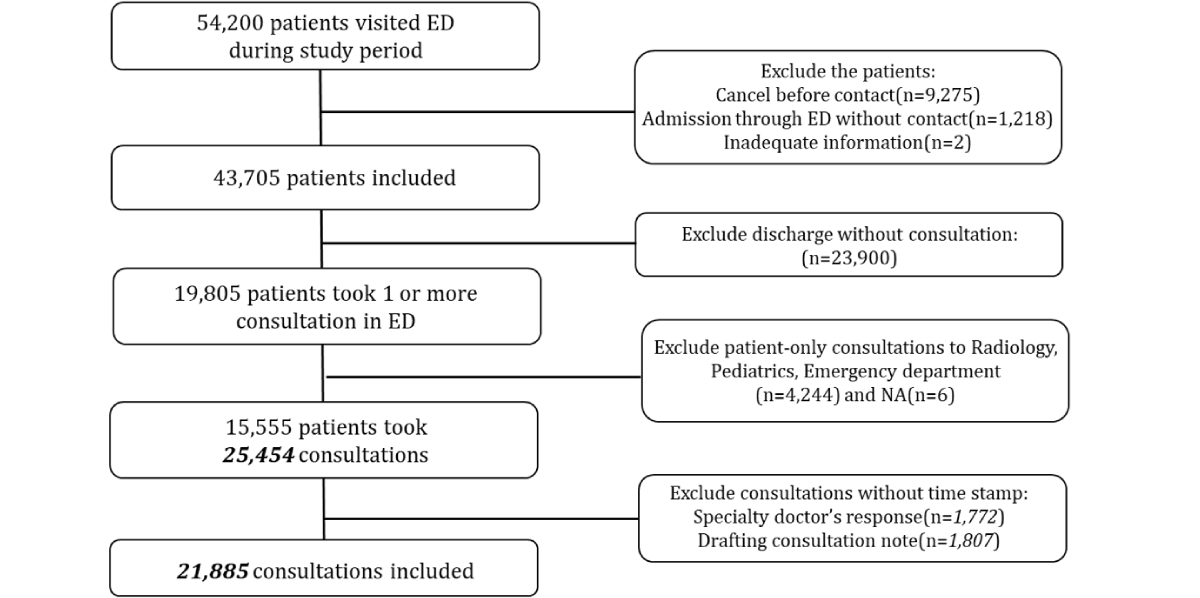
Flowchart of patient inclusion in the specialty consultation process. ED: emergency department; NA: No information about consultation department.

A total of 54,200 patients visited the ED during the study period. After excluding patients based on the exclusion criteria, 25,454 emergency consultations of 15,555 ED patients were included in the analysis. After excluding consultations lacking time information, 21,885 consultations were included in the analysis. Figure 3 presents the flowchart of subject inclusion. Multimedia Appendix 1 presents the demographics of the included subjects. Severity was assessed using the Korean Triage and Acuity Scale (KTAS), which was developed based on the Canadian Triage and Acuity Scale and is used across all Korean EDs. KTAS Level 1 indicates the highest acuity or severity of distress, and level 5 indicates the lowest [19].

**Figure 4:**
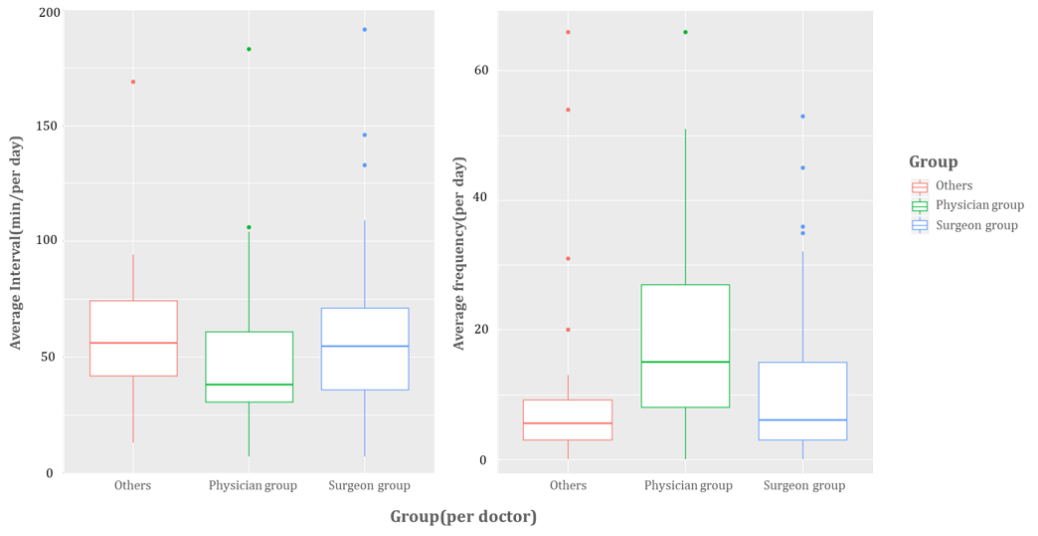
Median consultation completion interval and log frequency of doctors who used mobile electronic medical records in specialty consultation.

A total of 21,885 consultations were done, and 252 specialty doctors responded to these ED consultation requests. The specific departments of consultations are shown in Multimedia Appendix 2. The demographic characteristics of the doctors who provided consultations are shown in Table 1. Figure 4 provides mDARWIN usage patterns by group.

**Table 1:** Demographics of doctors related to specialty consultations.

Department	Position, n (%)	
	Staff and fellows	Resident
Physician group (n=86)	45 (52)	41 (48)
Surgeon group (n=120)	13 (11)	107 (89)
OHBP^a^ group (n=46)	19 (41)	27 (59)

^a^OHBP: other hospital-based physician.

### Log Data

During the study period, a total of 2,170,625 mobile EMR logs were created by 742 doctors of 39 specialty departments. Doctors included 218 professors (29%), 140 clinical fellows (19%), and 384 residents (52%). Of the 742 doctors, 208 used mobile EMR in the consultation process, and the following distribution was observed: physician group=34.6% (72/208), surgeon group=51.9% (108/208), and other hospital-based physician groups=13.5% (28/208). According to the mDARWIN usage pattern, the average log frequency of all users per day was 13.3, and the average time to complete the specialty consultation was 51.7 minutes. Among the three doctor groups, the physician group used mobile EMR more frequently (average usage=19.4) than the other groups, and the average period to complete the specialty consultation was the fastest at 45.3 minutes.

### Pearson’s Product-Moment Correlation

**Figure 5:**
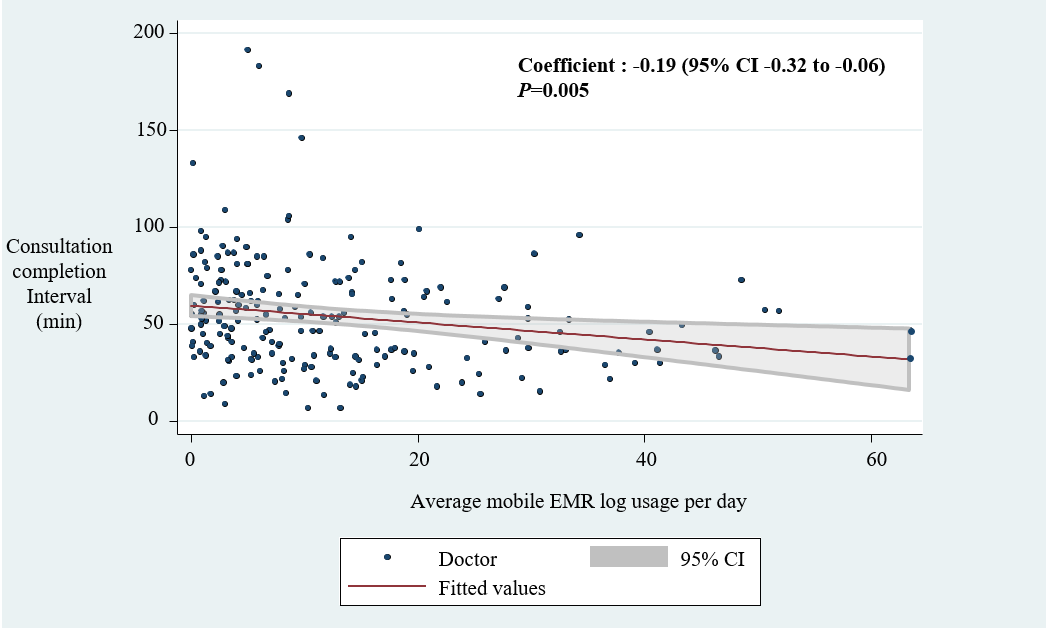
The correlation between consultation completion interval and mobile electronic medical record frequency. EMR: electronic medical record.

The results of the Pearson’s product-moment correlation (Figure 5) showed that consultation completion interval time had a significant inverse relationship with mobile EMR usage frequency (coefficient=–0.19; 95% CI –0.32 to –0.06; *P*=.005). Subgroup analysis classified specialty doctors who had left mobile logs in the consultation completion interval by frequency using the median. The median frequency was 2834, and completion time for the specialty doctors with high frequency (over 2834) was 78 minutes, while the completion time was 84 minutes for specialty doctors with low frequency. The difference was not statistically significant (Multimedia Appendix 3).

**Figure 6:**
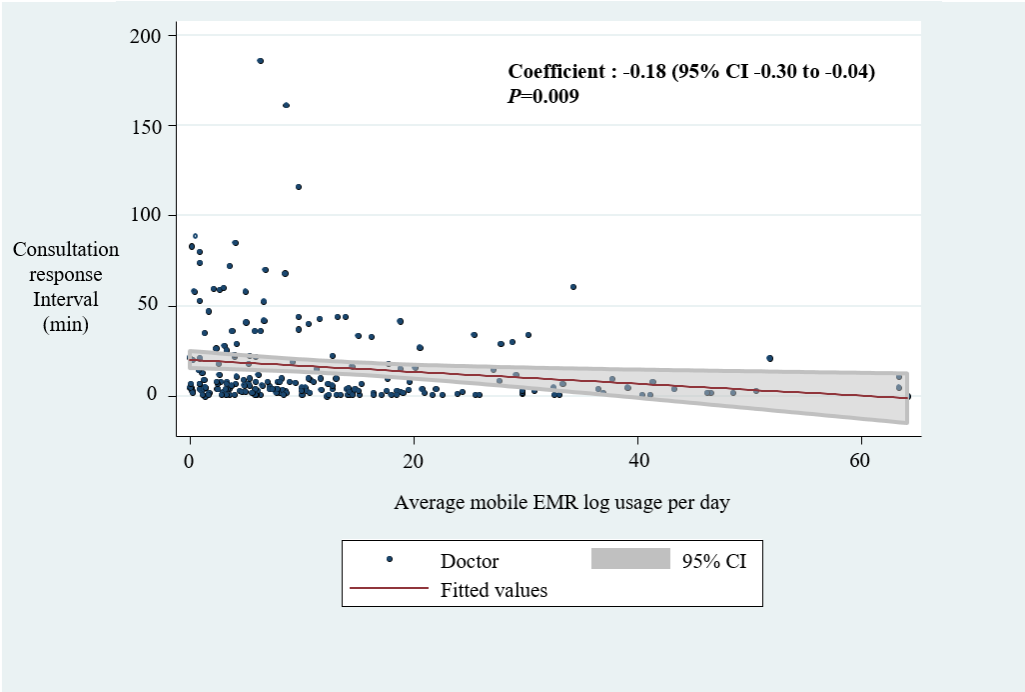
The correlation between consultation response interval and mobile electronic medical record frequency. EMR: electronic medical record.

The same analysis with different response interval times was performed to analyze the secondary outcome. There was a significant inverse relationship between the response interval time and mDARWIN usage frequency (coefficient=–0.18; 95% CI –0.30 to –0.04; *P*=.009) (Figure 6).

## Discussion

### Principal Findings

This study aimed to explore the association between mobile EMR usage and specialty consultation time based on mobile EMR log data and EMR timestamp data. Our findings indicate that specialty doctors using mobile EMR frequently responded to ED consultation requests quicker and answered ED consultations with less time than the less frequent users (Figure 5 and Figure 6). This result implies that mobile EMR might be helpful in effectively managing consultation delays.

To ensure consistency in the results, we performed the subanalysis with specialty doctors who left log records between receiving the request and drafting the consultation note. Our findings showed consistent results on the association between time interval and mobile EMR usage frequency, for both primary and secondary outcome measures, without statistical insignificance (Appendix 3).

Because ED consultations are unpredictable, the specialty physician’s actions after recognizing a consultation request vary from immediate to delayed, due to the demands of their daily jobs. Some specialty physicians could respond immediately and concentrate only on the ED consultation, while others inevitably dealt with ED consultations and other jobs simultaneously. In the analysis of real-world retrospective data, it is difficult to know whether all log data are related to ED consultation, even if log data appears in the interval between consultation requests and drafting consultation notes. Instead of cutting log data, we analyzed the total log data frequency. Nevertheless, the steps “response to request” and “drafting consultation note” after concluding the consultation process are unchangeable. For this reason, we analyzed the intervals between steps.

### Mobile Electronic Medical Records in Real Emergency Department Flow

ED crowding is generally estimated by length of stay [20], and length of stay is affected by three factors: input, throughput, and output factors [21]. Delayed response to ED consultation is a crucial independent variable of throughput and output factors of ED overcrowding conceptualization [1,22]. In the initial stages of the implementation of computerized EMR, text messaging and the use of a consultation management application were suggested, both of which were efficient [7,8]. Considering the accelerated use of mobile EMR in the present times, this study suggests the effectiveness of ED consultations.

The adoption of a mobile device in health care practice has generally contributed to improvement in clinical workflow, timeliness of communication, and patient safety [15,23-25]. A previous study reported that mobile communication using WhatsApp messenger could reduce consultation delays [26]. However, to the best of our knowledge, this study is the first to reveal that workflow efficiency and mobile EMR usability, represented by response interval and log frequency, are positively related to real-life ED settings. The improvement might be attributable to the features of mobile technology, such as the immediate alarm function in the mobile device and ubiquitous accessibility to mobile EMR. Before the mobile EMR alarm system, most of the communication about ED consultation was done via text message and phone calls. Notification of consultation via mobile EMR has helped eliminate redundant communication and deliver the patient information in the mobile EMR itself. While communication through mobile messenger has a potential risk to the security of the patient’s information when using personal devices [27,28], mobile EMR with access restriction is a much safer alternative for preventing confidentiality breaches.

As the contents of the mobile EMR to cover patient information is narrower than that of the EMR, it is important to consider the information covered in the mobile EMR. Different doctors and tasks require different contents [29]. Heavy information might result in fatigue, and light information might make the notification useless. Summarized patient information should be emphasized.

A previous study pointed out that doctors tended to underestimate mobile EMR objective improvement in response time, which resulted in its lower usability [15]. Of the 252 doctors included in this study, 17.4% of doctors (44/252) never used mobile EMR to manage consultations. There were several reasons for using only PC, such as not using an Android phone, unfamiliarity with the device, or not being aware of mobile EMR. It is difficult to determine the reasons for doctors not using mobile devices in this study. Surveying the nonuser group is a key to enhancing the usability of mobile EMR.

### Limitations

This study has some limitations. First, as this study was a single-center and single-department study of its system, the findings have limited generalizability. However, consultation delay remains a challenge, with many limitations that need to be addressed [8,30]; the use of the mobile EMR system has been shown to be valuable in terms of evidence.

Second, because the results of this study were based on an analysis of mobile log data, the reasons for usage patterns were not considered. An additional mixed-methods analysis, such as a user interview or survey, might better explain the usefulness of a mobile EMR system in the consultation process [31], and further evaluation of its usability might provide a clearer picture.

Third, we could not determine the causality of log data because we analyzed the entire log dataset. Although the entire log sufficed to achieve the study aim, an observational study using the small cut log of mobile EMR would be needed to demonstrate the association between behavior and mobile EMR usage.

### Conclusion

Our findings suggest that frequent usage of mobile EMR is associated with improvement in ED consultation delays.

## Appendix

Multimedia Appendix 1 Demographic characteristics of the patients receiving specialty consultations.

Multimedia Appendix 2 Number of consultation cases by department.

Multimedia Appendix 3 Comparison between high and low frequency users among specialty doctors who had left log records during the consultation completion interval.

## Abbreviations

DARWIN: Data Analysis & Research Window for Integrated kNowledge
ED: emergency department
EMR: electronic medical record
KTAS: Korean Triage and Acuity Scale
mDARWIN: mobile Data Analysis & Research Window for Integrated kNowledge
