# Correction: Frequent Mobile Electronic Medical Records Users Respond More Quickly to Emergency Department Consultation Requests: Retrospective Quantitative Study

**DOI:** 10.2196/43829

**Published:** 2022-11-08

**Authors:** Kwang Yul Jung, SuJin Kim, Kihyung Kim, Eun Ju Lee, Kyunga Kim, Jeanhyoung Lee, Jong Soo Choi, Mira Kang, Dong Kyung Chang, Won Chul Cha

**Affiliations:** 1 Department of Emergency Medicine Inha University School of Medicine Incheon Republic of Korea; 2 Department of Digital Health Samsung Advanced Institute for Health Science & Technology Sungkyunkwan University Seoul Republic of Korea; 3 Korea Health Industry Development Institute Cheongju Republic of Korea; 4 Statistics and Data Center Research Institute for Future Medicine Samsung Medical Center Seoul Republic of Korea; 5 Health Information and Strategy Center Samsung Medical Center Seoul Republic of Korea; 6 Center for Health Promotion Samsung Medical Center Sungkyunkwan University School of Medicine Seoul Republic of Korea; 7 Department Gastroenterology Samsung Medical Center Sungkyunkwan University School of Medicine Seoul Republic of Korea; 8 Department of Emergency Medicine Samsung Medical Center Seoul Republic of Korea

In “Frequent Mobile Electronic Medical Records Users Respond More Quickly to Emergency Department Consultation Requests: Retrospective Quantitative Study” (JMIR Mhealth Uhealth2020;8(2):e14487) the authors noted one error.

In the originally published article, an affiliation of author Kwang Yul Jung (Affiliation 2) was inadvertantly excluded.

The corrected version now indicates both the following affiliations of author Kwang Yul Jung:

^1^Department of Emergency Medicine, Inha University School of Medicine, Incheon, Republic of Korea

^2^Department of Digital Health, Samsung Advanced Institute for Health Science & Technology, Sungkyunkwan University, Seoul, Republic of Korea

The correction will appear in the online version of the paper on the JMIR Publications website on November 8, 2022, together with the publication of this correction notice. Because this was made after submission to PubMed, PubMed Central, and other full-text repositories, the corrected article has also been resubmitted to those repositories.

