# Supplementary material for: Frequent Mobile Electronic Medical Records Users Respond More Quickly to Emergency Department Consultation Requests: Retrospective Quantitative Study
Source: JMIR Mhealth Uhealth. 2020 Feb 14;8(2):e14487. doi: 10.2196/14487 (PMC7055754; doi:10.2196/14487)
Supplement: Multimedia Appendix 1 [file mhealth_v8i2e14487_app1.pdf]

Multimedia Appendix 1. Demographic characteristics of the patients receiving specialty consultations

**Variables**		N	%
**Total patients**		**14,225**	**100**
**Gender**	Male	7,460	52.4
	Female	6,765	47.6
**Age**	< 19	1,416	10
	19 ~ 64	7,445	52.3
	> 65	5,364	37.7
**Total consultation cases (visit cases)**		**21,885**	**100**
**KTAS[a] level**	Level 1	128	0.6
	Level 2	1,886	8.6
	Level 3	11,688	53.4
	Level 4	7,438	34
	Level 5	745	3.4
**Visit reason**	Disease	18,852	86.1
	Injury	3,028	13.8
	Others	5	0.1
**Visit path**	Direct ED visit	16,000	73.1
	From other hospital	4,926	22.5
	From outpatient department	940	4.3
	Others	19	0.1
**Visit method**	Ambulatory	15,345	70.1
	EMS[b]	3,524	16.1
	Others	3,004	13.8

[a]KTAS: Korean triage and acuity scale.

[b]EMS: emergency medical system.
