# Supplementary material for: Frequent Mobile Electronic Medical Records Users Respond More Quickly to Emergency Department Consultation Requests: Retrospective Quantitative Study
Source: JMIR Mhealth Uhealth. 2020 Feb 14;8(2):e14487. doi: 10.2196/14487 (PMC7055754; doi:10.2196/14487)
Supplement: Multimedia Appendix 2 [file mhealth_v8i2e14487_app2.pdf]

Multimedia Appendix 2. Number of consultation cases by department.

Group	%	Department	Consultation(N)
**Physician group**	**42**	Gastroenterology	1,365
		Cardiology	1,087
		Pulmonology	674
		Endocrinology	98
		Nephrology	458
		Hemato-oncology	581
		Infection	811
		Allergy	27
		Rheumatology	67
		Internal medicine	3,976
**Surgeon group**	**45**	Otolaryngology	1,689
		Surgery	1,878
		Neurosurgery	1,165
		Obstetrics and gynecology	877
		Ophthalmology	1,079
		Orthopedics	1,415
		Plastic surgery	734
		Thoracic surgery	429
		Urology	672
**OHBP[a] Group**	**13**	Critical Care Medicine	409
		Dental	170
		Neurology	1,894
		Psychiatry	519
		Rehabilitation	9
		Radiation Oncology	9
			**21,885**

[a]OHBP: other hospital-based physician.
