# Supplementary material for: Frequent Mobile Electronic Medical Records Users Respond More Quickly to Emergency Department Consultation Requests: Retrospective Quantitative Study
Source: JMIR Mhealth Uhealth. 2020 Feb 14;8(2):e14487. doi: 10.2196/14487 (PMC7055754; doi:10.2196/14487)
Supplement: Multimedia Appendix 3 [file mhealth_v8i2e14487_app3.pdf]

Multimedia Appendix 3. Comparison between high and low frequency users among specialty doctors who had left log record during the consultation completion interval.

	**High frequency users (>2,834)**	**Low frequency users (<2,834)**	***P* value**
Median	78min	84min	0.26
Mean	81min(±34.3)	85min(±40.04)	

High frequency user criteria: Median of Frequency (2,834)
